# Prognostic Implication of the Expression Level of PECAM-1 in Non-small Cell Lung Cancer

**DOI:** 10.3389/fonc.2021.587744

**Published:** 2021-03-22

**Authors:** Shuhui Cao, Yue Wang, Jingwen Li, Xuxinyi Ling, Yao Zhang, Yan Zhou, Hua Zhong

**Affiliations:** Department of Pulmonary, Shanghai Chest Hospital, Shanghai Jiaotong University, Shanghai, China

**Keywords:** non-small cell lung cancer, single-cell RNA-seq, gene expression omnibus database (GEO), the Cancer Genome Atlas (TCGA), PECAM-1 (CD31)

## Abstract

**Background:** Lung cancer is a malignant disease that threatens human health. Hence, it is crucial to identify effective prognostic factors and treatment targets. Single-cell RNA sequencing can quantify the expression profiles of transcripts in individual cells.

**Methods:** GSE117570 profiles were downloaded from the Gene Expression Omnibus database. Key ligand-receptor genes in the tumor and the normal groups were screened to identify integrated differentially expressed genes (DEGs) from the GSE118370 and The Cancer Genome Atlas Lung Adenocarcinoma databases. DEGs associated with more ligand-receptor pairs were selected as candidate DEGs for Gene Ontology (GO) functional annotation, Kyoto Encyclopedia of Genes and Genomes (KEGG) pathway analysis, and survival analysis. In addition, we conducted validation immunohistochemical experiments on postoperative specimens of 30 patients with lung cancer.

**Results:** A total of 18 candidate DEGs were identified from the tumor and the normal groups. The analysis of the GO biological process revealed that these DEGs were mainly enriched in wound healing, in response to wounding, cell migration, cell motility, and regulation of cell motility, while the KEGG pathway analysis found that these DEGs were mainly enriched in proteoglycans in cancer, bladder cancer, malaria, tyrosine kinase inhibitor resistance in Epidermal Growth Factor Receptor (EGFR), and the ERBB signaling pathway. Survival analysis showed that a high, rather than a low, expression of platelet endothelial cell adhesion molecule-1 (PECAM-1) was associated with improved survival. Similarly, in postoperative patients with lung cancer, we found that the overall survival of the PECAM-1 high-expression group shows a better trend than the PECAM-1 low-expression group (*p* = 0.172).

**Conclusions:** The candidate DEGs identified in this study may play some important roles in the occurrence and development of lung cancer, especially PECAM-1, which may present potential prognostic biomarkers for the outcome.

## Introduction

Lung cancer (LC) is the leading cause of cancer-related deaths among men and the third most common type of cancer among women, accounting for an estimated 2.1 million new cases and ~1.9 million deaths worldwide in 2018. Non-small cell LC (NSCLC) is the most common type of LC, accounting for about 85% of cases (1). Tumors of NSCLC typically undergo extensive genomic changes. Recently, molecularly targeted therapies and immune checkpoint inhibitors have dramatically improved the survival of patients with genomic changes to somatic cells (2). However, patients with LC often have different outcomes with the same therapy, and resistance to targeted therapies and immunotherapies remains problematic. Hence, the identification of a new biomarker of prognosis is needed.

Single-cell genomics is a powerful tool to explore genetic and functional heterogeneity, reconstruct evolutionary lineages, and detect rare subpopulations (3). Single-cell RNA sequencing (scRNA-seq) of human tumors has revealed new insights into tumor heterogeneity and the identification of different cell subpopulations, which are crucial to elucidate the mechanisms underlying tumorigenesis (4, 5). Furthermore, a better understanding of the gene expression profiles of the tumor microenvironment (TME) may help to improving prognosis and identifying molecular therapeutic targets.

Recently, intra-tumor mutational diversification analysis of LC at the single-cell level has been conducted (2, 6, 7). However, the scRNA-seq analysis has not yet been implemented to compare the gene expression profiles of non-small cell LC with those of normal tissues.

In the present study, the genomic features of LC cells and adjacent normal cells were obtained from GSE117570 and were analyzed to sort and screen key genes coding for ligand receptors. Then, candidate differentially expressed genes (DEGs) from The Cancer Genome Atlas Lung Adenocarcinoma (TCGA-LUAC) and GSE118370 databases were used for an enrichment analysis to identify those associated with crucial ligand-receptor activities to improve the efficacy of individualized treatment regimens for LC.

## Materials and Methods

### Patients

This analysis enrolled patients with newly diagnosed, pathologically confirmed NSCLC at the Shanghai Chest Hospital (Shanghai, China) from December 1, 2012 to December 31, 2017. All patients underwent complete resection, and no distant metastases were observed. All patients underwent follow-up of survival once a year. Paraffin-embedded lung adenocarcinoma specimens were obtained from all participants, and clinical, as well as pathological, data were collected. The present study was approved by the Institutional Review Board for Clinical Research of the Shanghai Chest Hospital.

### Data Curation

The GSE117570 and GSE118370 datasets were downloaded from the Gene Expression Omnibus database (https://www.ncbi.nlm.nih.gov/geo/query/acc.cgi). The expression profiles of LC samples were downloaded from TCGA-LUAD database (https://portal.gdc.cancer.gov/).

### Quality Control

The DropletUtils function of the R package was used to characterize the gene expression profiles of individual cells and to filter out any gene with counts of zero for all barcodes (8). Then, further screening was conducted of each cell with <100 unique molecular identifiers. The calculateQCMetrics scater package (9) was used to filter cells with ≤5% of mitochondrial genes and ≥10% of ribosomal genes. The expression matrix of each sample was normalized with the NormalizeData function included with the Seurat package (10).

### Principle Component Analysis (PCA) and *t*-Distributed Stochastic Neighbor Embedding (t-SNE)

The FindVariableFeatures function of the Seurat package was used to screen the top 2,000 genes with the highest standard deviations and defined as high variants. Focusing on high variant genes by downstream analysis helps to highlight biological signals in single-cell data sets. Then, the ScaleData function of the Seurat package was used to linearly scale the expression data. Finally, the RunPCA function of the Seurat package was used for linear dimensionality reduction analysis. After the selection of the principal components with large SDs, the FindNeighbors and FindClusters functions of the Seurat package were used for cell clustering analysis. Later, the RunTSNE function of the Seurat package was used for the non-linear dimensionality reduction analysis *via t*-SNE.

### Marker Gene Identification

The FindAllMarkers function of the Seurat package was used to identify DEGs between each cluster and other cell types [logFC ≥ 0.25 (expression ratio of the cell population ≥0.25); *p* ≤ 0.05] as marker genes. Cell clusters were labeled and visualized according to existing annotations in the CellMarker database (11).

### Screening of DEGs

Genes in TCGA-LUAD and the GSE118370 databases with a significant difference in mean values among all samples (ANOVA; *p* ≤ 0.05) were selected for PCA. Samples with appropriate phenotypes were selected for differential expression analysis with the use of GSE118370 chip data with the Limma package and of TCGA sequencing data with the edgeR package after log2 conversion of each sample (|log2FC| ≥ 0.5849625). Then, DEGs were collected.

### Ligand Receptor Network Analysis

Based on the ligand-receptor pairing data, related ligand-receptor pairs of various cell types were analyzed, counted, and organized by networks (12).

### Gene Function Enrichment Analysis

Functional enrichment analysis of DEGs was conducted with the use of the Gene Ontology (GO) and the Kyoto Encyclopedia of Genes and Genomes (KEGG) biochemical pathway databases (13, 14). The Fisher's exact test was used to determine the specific functions of DEGs. The *p*-value and the false discovery rate were calculated for each DEG. The smaller the *p-*value, the greater the relationship between the functional item and the input gene, as most DEGs in the same group had similar functions.

### Gene Set Enrichment Analysis (GSEA)

Based on the genes included in the GSE118370 database, GSEA was used to compensate for the deficiency of a single gene (15).

### Immunohistochemistry (IHC)

Anti-PEACM1 antibody (1:50, ab28364, Abcam) was used for IHC staining. After the staining was completed, two pathologists independently scored the stained samples according to the staining intensity and the percentage of positively stained cells. The staining intensity was scored as follows: 0 (no staining), 1 (yellow or yellow-brown), and 2 (brown). The percentage of positive cells was scored as follows: 0 (none), 1 (<10%), 2 (10–50%), and 3 (<50%). Then, the relative expression index was calculated by multiplying these two scores; the final score <3 indicates low expression, and the final score ≥ 3 indicates high expression.

### Survival Analysis

According to the TCGA database, we defined the median value of the expression of candidate DEGs in all patients as the cut-off value and performed the Kaplan-Meier Survival analysis and the COX regression analysis by using the survival package. Correlations among the characteristics of patients in different groups were analyzed with the Fisher's exact test and performed using the SPSS software version 22 (IBM Corporation, Chicago, IL).

## Results

### Cell Cluster Compositions of Tumor Tissues and Normal Tissues

**Figure 1:**
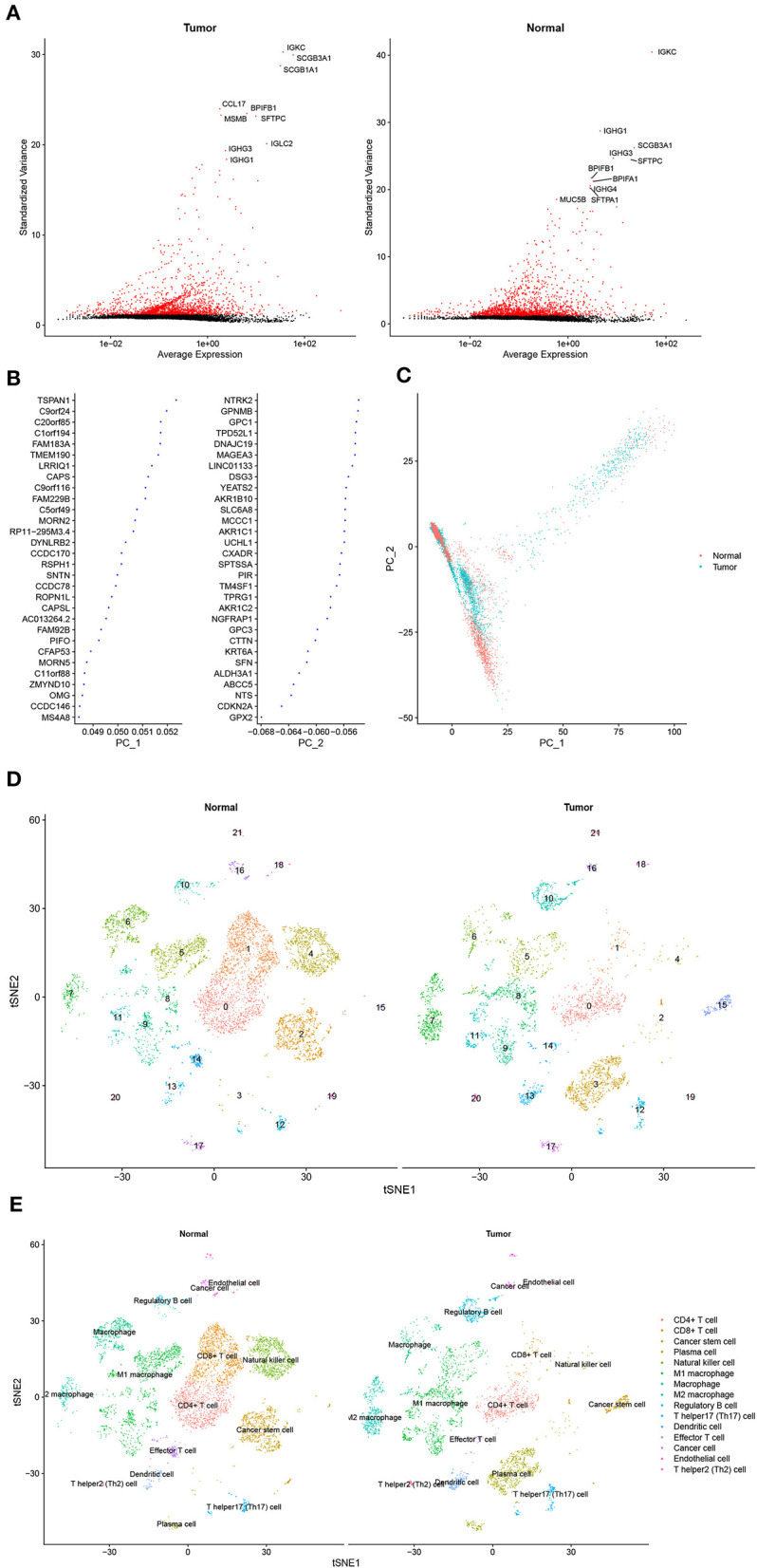
Cell cluster compositions of tumor tissues and normal tissues. **(A)** Top 10 genes with the most significant differences in SDs. **(B)** Gene contributions in two principal components (PC), namely PC_1 and PC_2. **(C)** Distribution of cells in two dimensions based on the PC_1 and PC_2 components. **(D,E)**. Cell clusters of *t*-distributed stochastic neighbor embedding (*t*-SNE) and identified marker genes.

Approximately, 11,233 cells from the GSE117570 database passed quality control and were selected for further analysis (Supplementary Figure 1A). The top 2,000 genes with the most significant differences in SDs were screened, and the top 10 genes are revealed in Figure 1A. The distribution of these genes between the tumor group and the normal group was detected by PCA and *t*-SNE. The top 46 significantly correlated genes are shown in Supplementary Figure 1B. We mapped the cells into two dimensions based on the PC_1 and PC_2 components, and other components were calculated with an estimated *p*-value, and the significant components were selected for subsequent analysis (Figures 1B,C). To further precisely cluster the populations of cells, *t*-SNE was adopted for the visualization of high dimensional data (Figure 1D). In total, 15 distinct cell clusters were identified by clustering analysis and classified based on the top 10 DEGs, which included CD4+T cells, CD8+ T cells, cancer stem cells, plasma cells, natural killer cells, M1 macrophages, macrophages, M2 macrophages, regulatory B cells, T helper 17 (Th17) cells, dendritic cells, effector T cells, cancer cells, endothelial cells, and Th2 cells (Figure 1E).

### Filtering and Functional Enrichment Analysis of DEGs

**Figure 2:**
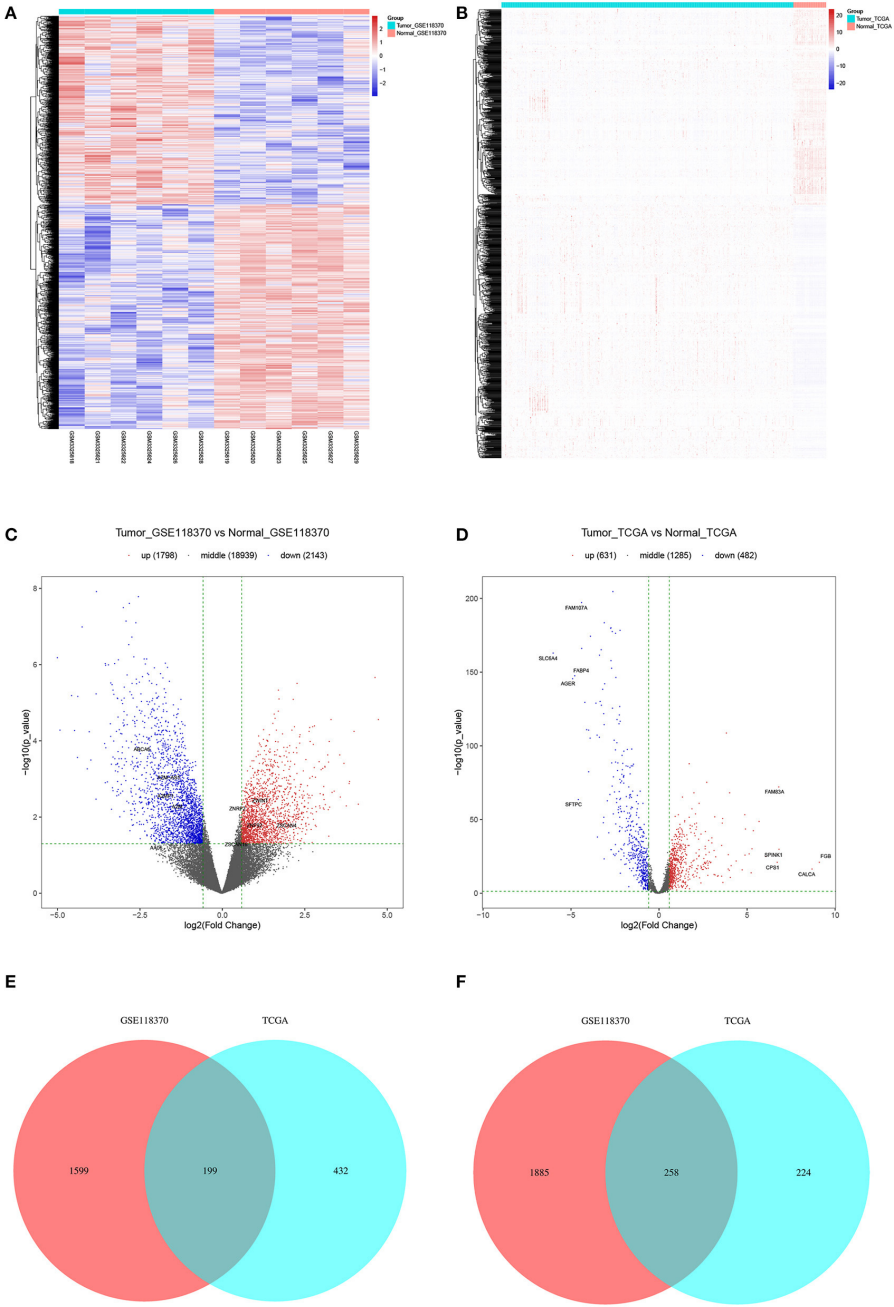
Filtering of differentially expression genes (DEGs). **(A,B)** Heat maps of DEGs of **(A)** GSE118370 and **(B)** The Cancer Genome Atlas (TCGA). **(C,D)** Volcano plot of DEGs of **(C)** GSE118370 and **(D)** TCGA. **(E,F)** The intersection of **(E)** upregulated DEGs and **(F)** downregulated DEGs of GSE118370 and TCGA.

**Figure 3:**
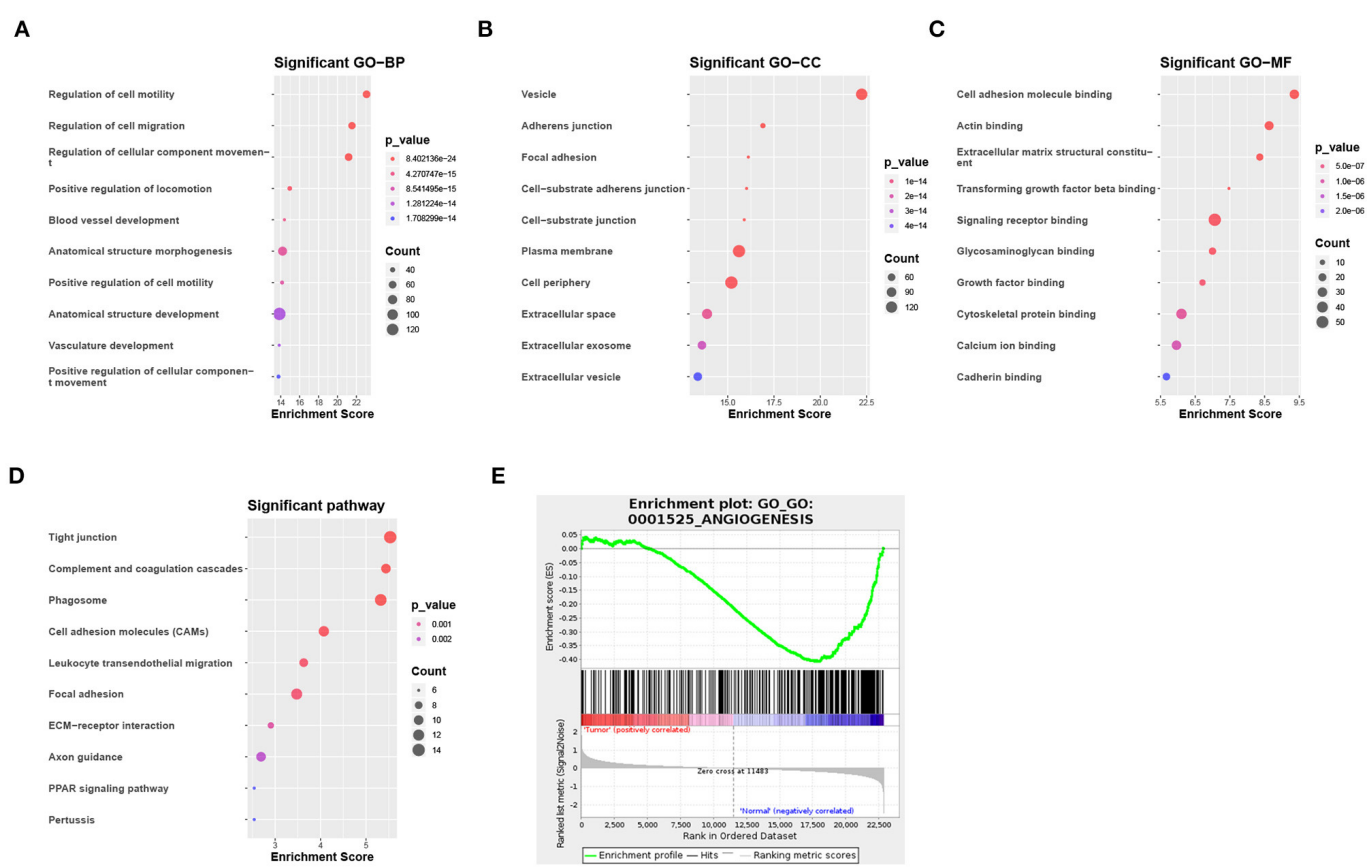
Functional enrichment analysis of DEGs. **(A–C)** Enrichment result of downregulated DEGs in **(A)** the biological process (BP), **(B)** the cellular component (CC), and **(C)** the molecular function (MF) pathway of Gene Ontology (GO). **(D)** Enrichment result of downregulated DEGs of the Kyoto Encyclopedia of Genes and Genomes (KEGG) pathway. **(E)** Gene set enrichment analysis result of GO:0001525 (ANGIOGENESIS).

Of the 3,941 DEGs identified in the GSE118370 dataset, 1,798 were up-regulated and 2,143 were downregulated (Figures 2A,C; Table 1). Of the 1,113 DEGs identified in the TCGA dataset, 631 were upregulated and 482 were downregulated (Figures 2B,D; Table 1). Of the 457 shared DEGs in the two databases, 199 were upregulated and 258 were downregulated (Figures 2E,F). To further investigate cell functional states associated with LC and potential molecular regulators, functional enrichment analyses, including GO and KEGG analyses, of these DEGs were conducted. Three main categories of the GO function analysis [biological process (BP), cellular component (CC), and molecular function (MF)] revealed that the downregulated DEGs were significantly enriched in the following functions: cell motility, cell migration, and cell component movement (GO BP, Figure 3A); vesicle (GO CC, Figure 3B); and cell adhesion molecule binding, actin binding, and extracellular matrix structural constituent (GO MF, Figure 3C). According to the results of the KEGG analysis, the downregulated DEGs were mainly enriched in tight junctions, complement and coagulation cascades, and phagosomes (Figure 3D).

**Table 1:** Statistic result of differentially expressed genes(DEGs).

**Resources**	**Platform**	**Sample size of Tumor**	**Sample size of Normal**	**FC**	***P*_value**	**Up**	**Down**
GSE118370	GPL570	6	6	1.5	0.05	1,798	2,143
TCGA	HiSeq	526	59	1.5	0.05	631	482

To identify the potential functions of the DEGs in the tumor group and the normal group, GSEA was conducted to search GO terms enriched in the GSE118370 dataset (Figure 3E). The results showed that some of the genes expressed in the normal group were significantly and negatively correlated with the angiogenesis pathway (GO: 0001525).

### Ligand Receptor Network Analysis

**Figure 4:**
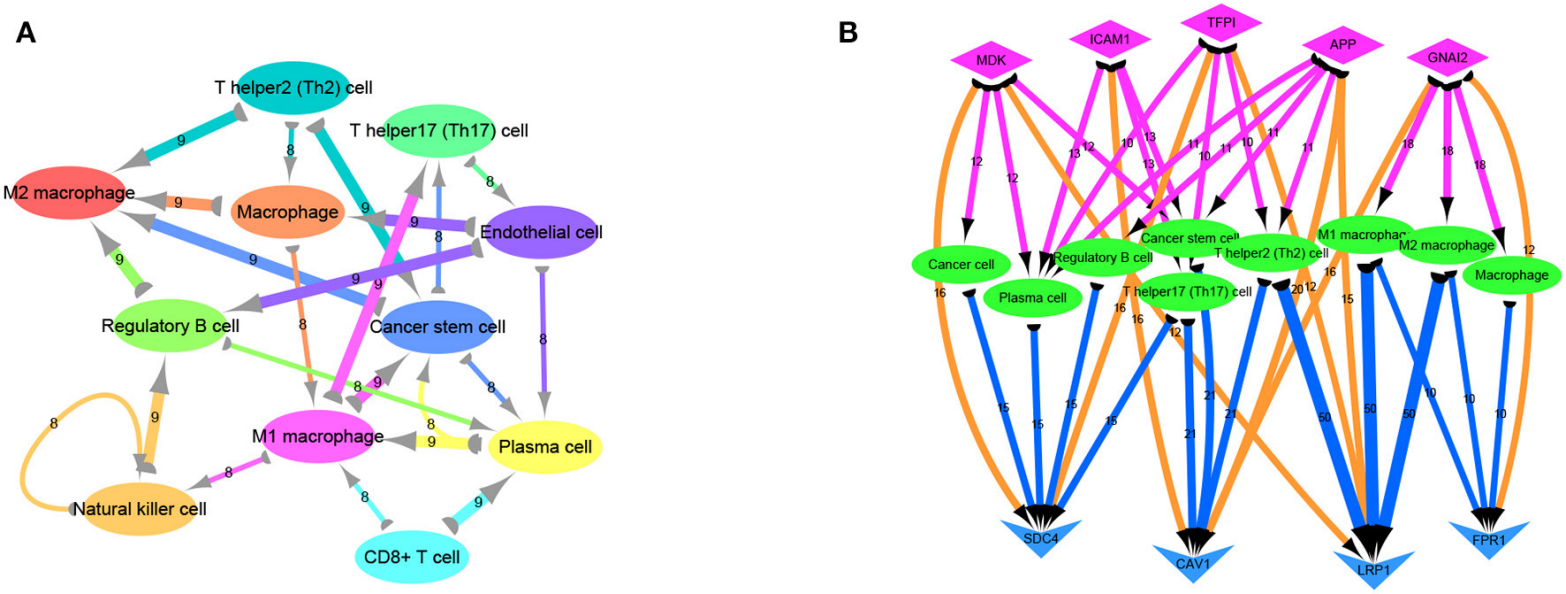
Ligand receptor network analysis. **(A)** Network diagram of the detailed relationship between ligand receptors in various cell types. **(B)** Based on the network pairing relationship, the top none most ligand-receptor relationship pairs are selected, and the number of relationships between them with cell group is counted, including SDC4-MDK (16 pairs); SDC4-TFPI (16 pairs); LRP1-MDK (12 pairs); CAV1-ICAM1 (16 pairs); CAV1-APP (20 pairs); CAV1-GNAI2 (16 pairs); LRP1-TFPI (12 pairs); LRP1-APP (15 pairs); FPR1-GNAI2 (12 pairs). Diamond-shaped nodes represent ligands, and the arrows from ligands to cell types means V-shaped nodes represent receptors.

Previous analyses of the ligand-receptor relationships of all marker genes in each cell type were presented as arrow diagrams (Supplementry Figure 1D; Figure 4A) (11). Finally, screening of nine ligand-receptor pairs with the most interactions identified a distinct network in individual cells (Figure 4B).

### Selection and Enrichment Analysis of Candidate DEGs

**Figure 5:**
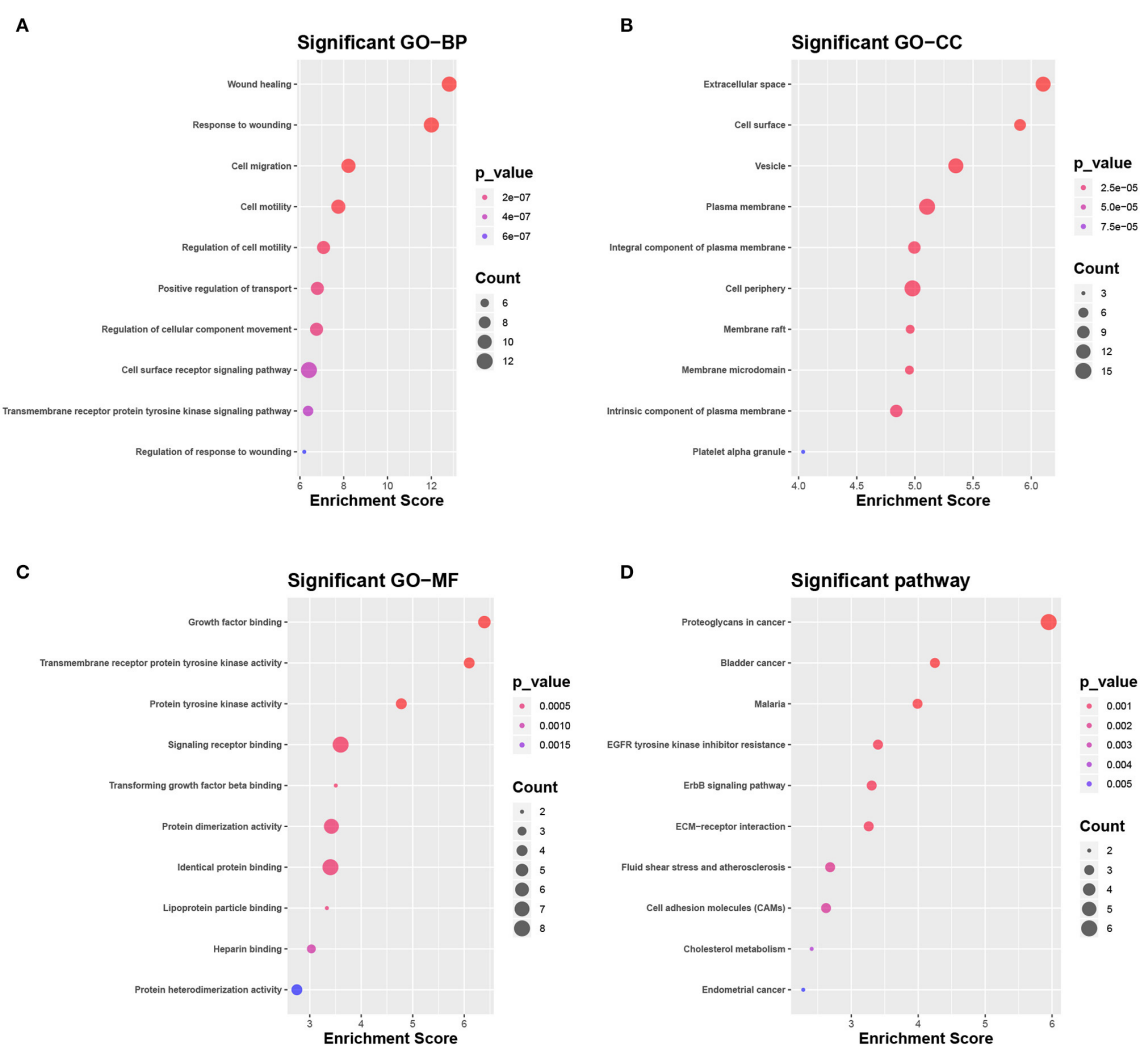
Selection and enrichment analysis of candidate DEGs. **(A–C)** Gene function enrichment analysis of candidate DEGs in **(A)** GO BP, **(B)** GO CC, and **(C)** GO MF. **(D)** Gene function enrichment analysis of candidate DEGs in the KEGG pathway.

The DEGs were compared with screened ligand-receptor pairs and the following top 18 transcripts were selected as candidate DEGs: AXL, C1QA, CAV1, CD36, CD93, CDH1, COL1A1, DDR1, EFNB2, ERBB2, ERBB3, GNAI2, HBEGF, LPL, MDK, PECAM-1, PROS1, and SDC1. Compared to normal tissues, CDH1, COL1A1, DDR1, ERBB2, ERBB3, MDK, and SDC1 were upregulated in LC, and AXL, C1QA, CAV1, CD36, CD93, EFNB2, GNAI2, HBEGF, LPL, PECAM-1, and PROS1 were downregulated. According to the GO analysis, the candidate DEGs were significantly enriched in wound healing, response to wounding, and cell migration (BP, Figure 5A), extracellular space, cell surface, and vesicle (CC, Figure 5B), and growth factor binding, transmembrane receptor protein kinase activity, and protein tyrosine activity (MF, Figure 5C). According to KEGG analysis, the candidate DEGs were significantly enriched in proteoglycans in cancer, bladder cancer, malaria, tyrosine kinase inhibitor resistance in the EGFR, the ERBB signaling pathway, ECM-receptor interactions, fluid shear stress and atherosclerosis, cell adhesion molecules, and cholesterol metabolism in endometrial cancer (Figure 5D).

### Survival Analysis of Candidate DEGs

**Figure 6:**
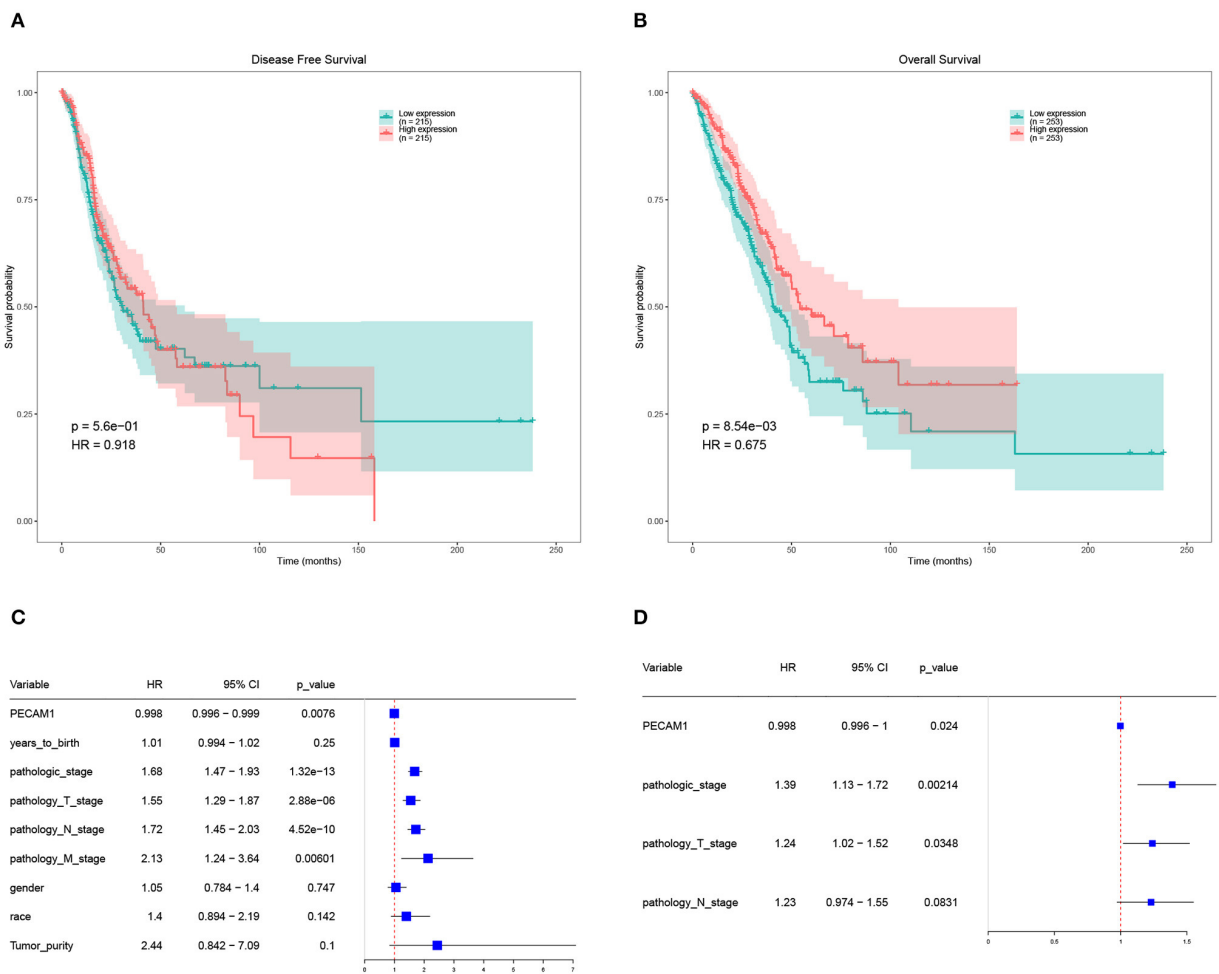
Survival analysis of PECAM-1. **(A)** Disease-free survival (DFS) of PECAM-1 expression. **(B)** Overall survival (OS) of PECAM-1 expression. **(C)** Univariate cox analysis of PECAM-1. **(D)** Multivariate cox analysis of PECAM-1.

Further analysis of the expression and clinical information of candidate DEGs in TCGA-LUAD database. We performed survival analysis on half of the patients with high expression levels and half of the patients with low expression levels of candidate DEGs and found that the expression of PECAM-1 had a significant impact on the survival of patients with LC at the threshold expression value of 8,615 (Figures 6A–D).

High expression of PECAM-1 apparently lead to a longer overall survival than its low expression [*p* = 0.00854, hazard ratio (HR) = 0.675, Figure 6B]. Univariate Cox analysis showed that PECAM-1 is a protective factor [HR = 0.998, 95% confidence interval (95%CI) = 0.996–0.999, *p* = 0.0076, Figure 6C]. Multivariate analysis was used for factors found to be obviously significant in univariate analysis, and the results of multivariate Cox analysis showed that PECAM-1 tended to have a protective effect (HR = 0.998, 95%CI = 0.996–1, *p* = 0.0024, Figure 6D).

### Validation of the Prognostic Effect of PECAM-1

**Figure 7:**
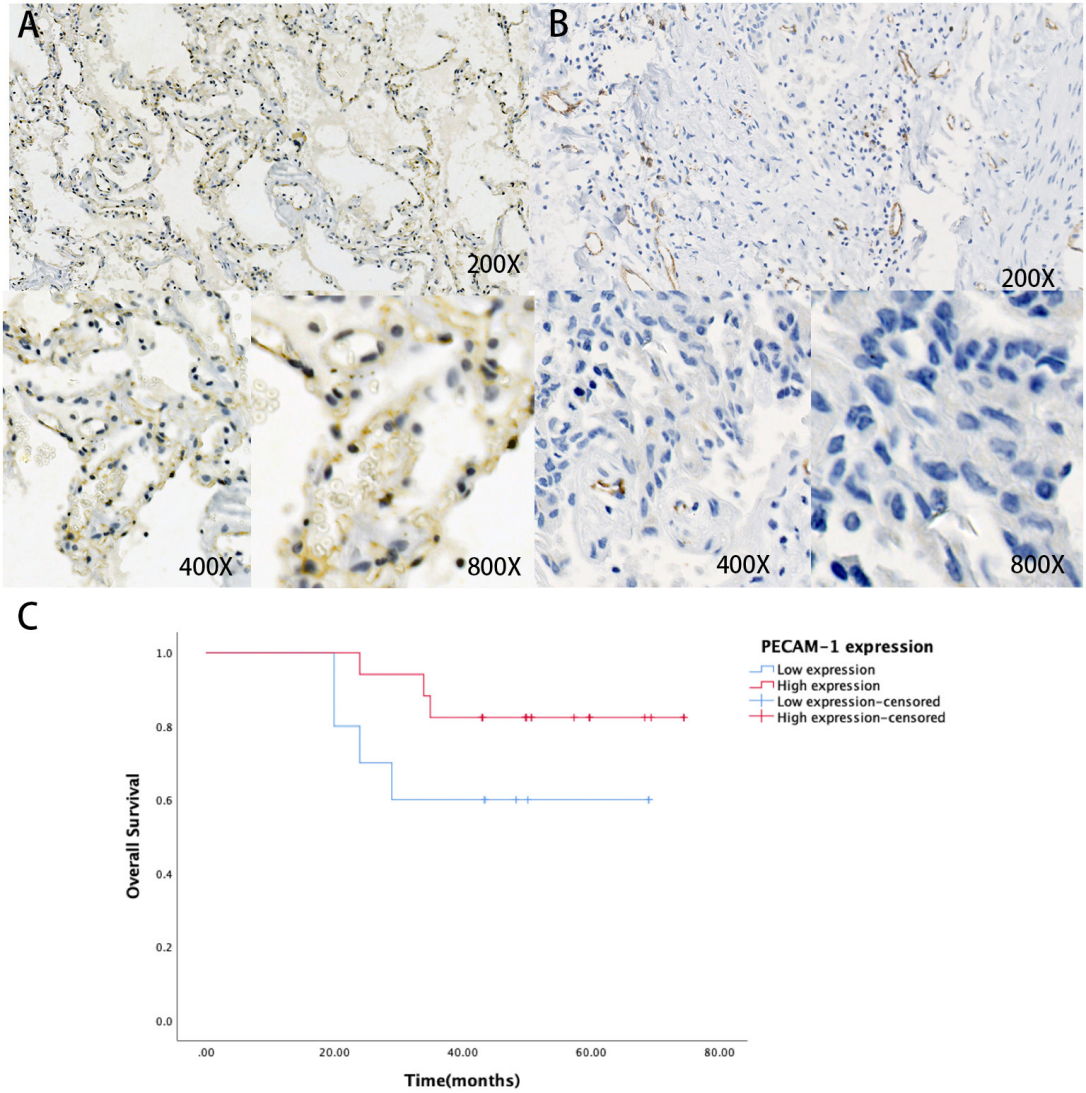
The expression of protein PECAM-1 in patients with lung adenocarcinoma. A-B Representative positive **(A)** and negative **(B)** expression of PECAM-1 were examined by immunohistochemistry in lung adenocarcinoma tissues (*n* = 30). Original magnification, ×200 (upper panel), ×400 (lower panel), ×800 (lower panel). **(C)** The association of protein expression of PECAM-1 with OS (*n* = 30).

First, we collected the paraffin tissue and clinical data of 30 patients with postoperative LUAC (Table 2). Then, we performed immunohistochemical tests to validate the expression of PECAM-1 in those paraffin specimens (Figures 7A,B). The median follow-up time is 50 months (three patients were lost). The Kaplan–Meier survival analysis of the overall survival of two groups showed that the PECAM-1 high-expression group showed a better survival trend than the PECAM-1 low-expression group, similar to our previous analysis (*p* = 0.172, Figure 7C).

**Table 2:** Demographic, clinical, and pathological characteristics of patients with lung adenocarcinoma in the PECAM-1 high-expression group (PECAM-1+) and the PECAM-1 low-expression group (PECAM-1-).

**Parameters**	**PECAM-1 +**	**PECAM-1 −**	***p*-value**
**Age**			1.000
≥60	6	5	
<60	11	8	
**Gender**			0.700
Male	10	9	
Female	7	4	
**Smoking history**			1.000
Non-smoker	16	13	
Smoker	1	0	
**Lymph node metastasis**		1.000
Yes	8	7	
No	9	6	
**TNM stage**			0.783
I	6	3	
II	5	3	
III	5	6	
IV	1	1	

## Discussion

In the present study, cell clusters in the tumor group and the normal group were identified. Screening of the top 18 candidate DEGs in two groups identified those expressed predominantly in ligand-receptor pairs with many interactions. Functional enrichment and survival analyses indicated that the candidate DEGs were significantly associated with the prognosis of LC, especially PECAM-1.

Overall, 18 candidate DEGs that participate in many ligand-receptor activities were subjected to scRNA-seq. The GO BP analysis results revealed that these DEGs were mainly enriched in the following top five functions: wound healing, response to wounding, cell migration, cell motility, and regulation of cell motility. Previous studies have demonstrated that the stroma of solid tumors contains a variety of cellular phenotypes and signaling pathways associated with wound healing. For example, tumor stroma is formed by abnormal activation of the wound healing pathways (16, 17). Both wound healing and TME are dependent on changes to deposition of the extracellular matrix, which promotes epithelial–mesenchymal transition and increases the motility of both fibroblasts and tumor cells (18). The GO CC analysis indicated that candidate DEGs enriched in extracellular space, cell surface, and vesicles may have important impacts on exosome production and tumor metastasis (19). The GO MF analysis demonstrated that some of the candidate DEGs were enriched in transmembrane receptor protein kinase activity and protein tyrosine activity, suggesting that the difference in the cellular processes of tumor cells vs. normal cells, such as cell signaling, cell-cell communication, transport, energy transduction, and enzyme activation, may be induced by receptor protein kinases. (20). These results suggest that these DEGs are involved in the establishment of the TME and the migration of LC cells.

The KEGG pathway analysis showed that the identified DEGs were mainly enriched in the following top five pathways: proteoglycans in cancer, bladder cancer, malaria, tyrosine kinase inhibitor resistance in EGFR, and the ERBB signaling pathway. Proteoglycans exert diverse functions in the occurrence of cancer (21–23) and are thought to regulate the phenotype of tumor cells and angiogenesis in tumor metabolism, in addition to promoting the formation of a temporary matrix for tumor growth, thereby affecting cell-cell interactions and cell-matrix interactions and tumor cell signal transduction (21). EGFR and the ERBB pathway are common targets for the treatment of LC (24, 25). The results of the present study showed DEGs have impact on tyrosine kinase inhibitor resistance in EGFR and the ERBB signaling pathway, which provide interesting insights for future studies of tyrosine kinase inhibitors in LC.

There were a lot of research about the 18 candidate DEGs. Expression of CDH1 (E-Cadherin) is associated with physiological signaling pathways, such as cell proliferation, maintenance of cell adhesion, cell polarity, and epithelial–mesenchymal transition. It is considered a risk factor for diffuse gastric and lobular breast cancer (26). COL1A1 expressions are found in most connective tissues and are abundant in bones, corneas, the dermis, and tendons (27). DDR1 is predominantly expressed in epithelial cells and is reported to be involved in the progression of cancer (28). Amplification of ERBB2 is well-described in many kinds of solid cancer and has been established as an important actionable target in multiple cancer types (29). ERBB3 plays an important role in cancer, and the mutation of ERBB is a potential tumor driver (30). MDK is a heparin-binding growth factor and acts as a mediator for the acquisition of critical hallmarks of cancer, including cell growth, survival, metastasis, migration, and angiogenesis (31). The expression of SDC1 often produces malignant phenotypes, which arise from increased cell proliferation and cell growth, cell survival, cell invasion and metastasis, and angiogenesis (32). AXL is a receptor tyrosine kinase expressed in many cancer types and has been associated with therapy resistance and poor clinical prognosis and outcomes (33). C1QA encodes the A-chain polypeptide of serum complement subcomponent C1q, which is associated with lupus erythematosus and glomerulonephritis (34). CAV1 encodes the scaffolding protein, which is the main component of the caveolae plasma membranes found in most cell types (35). CD36 is a scavenger receptor expressed in multiple cell types, and it mediates lipid uptake, immunological recognition, inflammation, molecular adhesion, and apoptosis (36). CD93 is a transmembrane receptor that is upregulated in tumor vessels in many types of cancer, including high-grade glioma (37). EFNB2 is expressed at abnormally high levels in some neoplasms, such as squamous cell carcinoma of the head and neck and colorectal cancer (38). The expression of GNAI2 in CD11c+ cells and IL6 in CD4+/CD11b+ DCs appears to promote colon tumor development in mice (39). HBEGF is a ligand for the EGFR, one of the most commonly amplified receptor tyrosine kinases in glioblastoma, which may be a clinically relevant target (40). LPL has been extensively investigated as a potential risk factor for coronary artery disease (41). PROS1 encodes a vitamin K-dependent plasma protein that functions as a cofactor for the anticoagulant protease, an activated protein C to inhibit blood coagulation. It plays an essential role in the resolution of inflammation (42).

PECAM-1 (also known as a cluster of differentiation 31, CD31) was primarily identified as an adhesion molecule that plays various roles in cell proliferation, apoptosis, and migration, in addition to cellular immunity. PECAM-1 is expressed by some tumor cells and may contribute to tumor invasion (43, 44). However, the role of PECAM-1 in LC remains unclear (45–50). Giovanna et al. found that PECAM-1 acts as a checkpoint molecule and can negatively regulate FcγR-mediated phagocytosis by monocytes and macrophages, and downregulation of PECAM-1 correlated with decreased survival of chronic lymphocytic leukemia cells (51, 52). Virman et al. found that high expression of PECAM-1 was significantly associated with improved survival.

At present, the research on PECAM-1 is not sufficient, but many studies have shown that PECAM-1 may affect immune regulation. This molecule may play a crucial and complex role in the regulation of T-cell-mediated immune responses, with a large impact on immunity in health and disease (53). Previous studies have found that although the loss of PECAM-1 leads to excessively cytotoxic killing, PECAM-1 also can delay T-cell apoptosis and prolong the action time of T cells (54, 55). Studies have also found that the PECAM-1 protein can promote the endothelial migration of lymphocytes and natural killer cell, which take pivotal roles in eliminating the abnormal cells, such as tumor cells (54, 56, 57). Analysis by our group revealed that a high PECAM-1 expression was associated with better overall survival and is a significant prognostic factor in LC. Although multivariate Cox analysis showed that PECAM-1 was not statistically significant, a suitable cutoff value of PECAM-1 expression may help to indicate better survival of patients with LC. Our research also found that, although it is statistically non-significant, patients with LC with a high PECAM-1 expression had a longer overall survival period, which may be related to the effect of PECAM-1 on the tumor immune microenvironment, promoting the transport of immune cells and enhancing the role of immune cells. However, the potential impact of PECAM-1-mediated interactions on the development and function of the immune system need to be fully studied.

The results of the present study identified key genes and pathways in LC, which will improve our understanding of the molecular mechanisms underlying the development and progression of LC. Eighteen genes that potentially play pivotal roles in the pathogenesis of LC and may be closely associated with tumor progression, especially PECAM-1, were identified in the present study. In addition, we conducted immunohistochemical analysis on the protein expression of PECAM-1 in lung cancer tissues and analyzed the survival of patients with LC with different PECAM-1 expressions. We found that the PECAM-1 high-expression group has a clear survival advantage, which further shows that PECAM-1 may be a protective prognostic factor.

## Data Availability Statement

Publicly available datasets were analyzed in this study. This data can be found here: the NCBI Gene Expression Omnibus (GSE117570 and GSE118370) and The Cancer Genome Atlas (https://portal.gdc.cancer.gov/) (TCGA LUAD).

## Ethics Statement

Written informed consent was obtained from the individual(s) for the publication of any potentially identifiable images or data included in this article.

## Author Contributions

HZ, YZho, and SC designed the study. SC and YW drafted the manuscript. SC, YW, and JL processed and analyzed data. XL and YZha edited the drafting of the manuscript and drew some pictures. HZ and YZho revised the manuscript. All authors contributed to the article and approved the submitted version.

## Conflict of Interest

The authors declare that the research was conducted in the absence of any commercial or financial relationships that could be construed as a potential conflict of interest.
