# Supplementary figures and images for: Prognostic Implication of the Expression Level of PECAM-1 in Non-small Cell Lung Cancer
Source: Front Oncol. 2021 Mar 22;11:587744. doi: 10.3389/fonc.2021.587744 (PMC8019905; doi:10.3389/fonc.2021.587744)

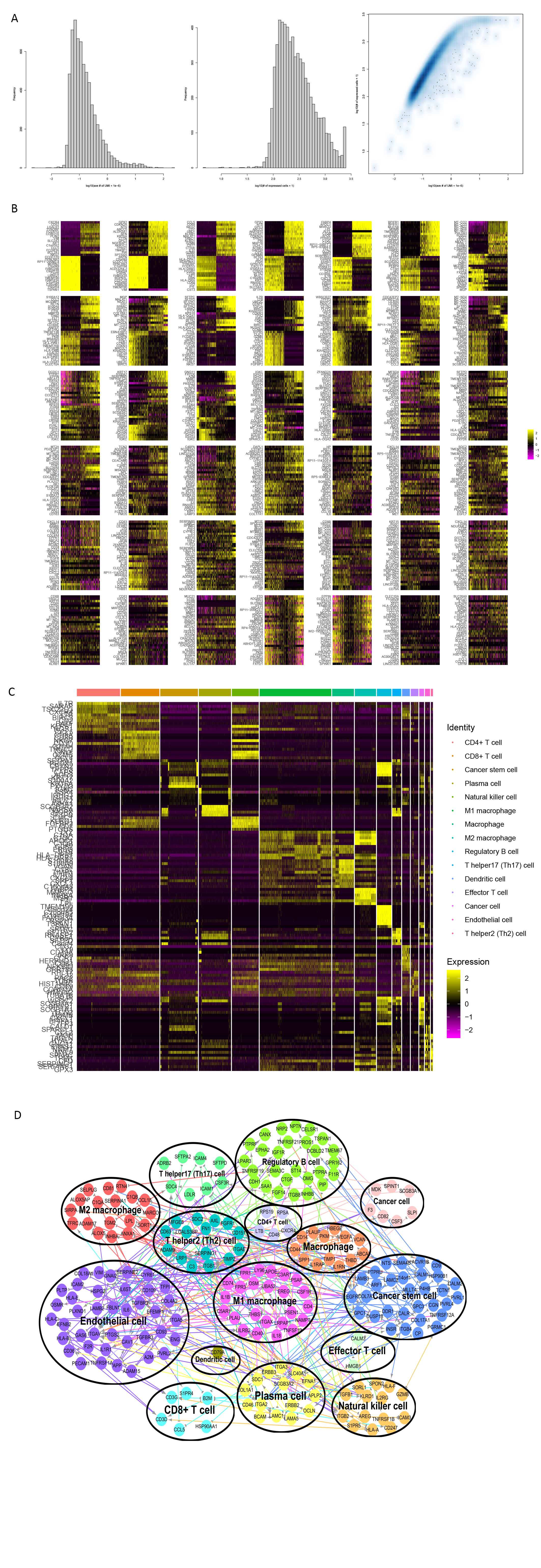

Supplement: Supplementary file 1 [file Image_1.TIF]
